# Deep Data Analysis-Based Agricultural Products Management for Smart Public Healthcare

**DOI:** 10.3389/fpubh.2022.847252

**Published:** 2022-04-07

**Authors:** Wenjing Yan, Zesheng Zhang, Qingchuan Zhang, Ganggang Zhang, Qiaozhi Hua, Qiao Li

**Affiliations:** ^1^National Engineering Laboratory for Agri-Product Quality Traceability, Beijing Technology and Business University, Beijing, China; ^2^Digital Campus Construction Center, Capital Normal University, Beijing, China; ^3^Computer School, Hubei University of Arts and Science, Xiangyang, China; ^4^Chongqing Key Laboratory of Intelligent Perception and Blockchain Technology, Chongqing Technology and Business University, Chongqing, China

**Keywords:** graph neural network, agricultural products, public healthcare, deep data analysis, smart management

## Abstract

Agricultural is an indispensably public healthcare industry for human beings at any time and smart management of it is of great significance. Since substantial technical advance relies on long-term efforts and continuous progress, reasonably scheduling the distribution of agricultural products acts as a key aspect of smart public healthcare. The most intuitive factor affecting the distribution of agricultural products is its dynamic price. Forecasting price fluctuations in advance can optimize the distribution of agricultural products and pave the way to smart public healthcare. Most researchers study the prices of various agricultural products separately, without considering the interaction of different agricultural products in the time dimension. This study introduces a typical deep learning model named graph neural network (GNN) for this purpose and proposes deep data analysis-based agricultural products management for smart public healthcare (named GNN-APM for short). The highlight of GNN-APM is to take latent correlations among multiple types of agricultural products into consideration when modeling evolving rules of price sequences. A case study is set up with the use of real-world data of the agricultural products market. Simulative results reveal that the designed GNN-APM functions well.

## 1. Introduction

Since ancient times, agriculture has been a life industry for human survival, which is closely related to the most basic life guarantee of human beings. At present, food shortage is one of the most important problems faced by many regions in the world (1). This phenomenon is generally reflected in two aspects (2). For one thing, there is still room for improvement in current agricultural technology, which makes grain yield fail to meet the expectations (3). For another, due to the lack of scientific management and scheduling strategy, the production of agricultural products is not reasonable (4). The exploration of advanced agricultural technology has lasted for at least a 100 years, and some technological breakthroughs have been made in some key fields (5, 6). However, this process exerts an imperceptible influence as we all know, which needs to be accumulated over a long period of time to make progress (7). The use of advanced computing technology to manage the agricultural products market can improve the distribution and dispatch efficiency of global agricultural products to a certain extent (8), and then alleviate the problem of food shortage (9). The key to improving management efficiency is to forecast the market conditions of several major types of agricultural products (10). To realize such a goal, data-driven methods are the most intuitive ways (11, 12).

Rationalizing the distribution of agricultural products is an important aspect of smart public healthcare. Predicting price fluctuations in advance can optimize the layout of agricultural products and pave the way for smart public healthcare. Many scholars have studied the agricultural products market in recent years (13–32). For example, Fan et al. (14) analyzed the value-added mechanism of agricultural products circulation value chain and put forward three optimization methods of agricultural products organization mode. These existing researches are mainly realized through the research methods of social sciences. Their analyses focus on the mechanism of social development and evolution but have to deal with large-scale computational tasks in a manual way. Without the assistance of intelligent computing, these methods are always faced with certain limitations. There are also some scholars who use intelligent computing methods to solve this problem. They regard the forecasting problem of the future agricultural products market as a time series forecasting problem based on historical data. However, most of them just treat different agricultural products as independent categories and then build time series prediction models separately. In this way, potential relationships between categories are ignored, so that the precision of the modeling process is reduced.

In order to solve the above challenges, a graph neural network (GNN) can be used to model this time series prediction problem (33). Different from the traditional model that deals with grid-structured data, GNN deals with the data of topological structure. GNN is based on deep learning (34) and is widely used in various fields due to its good performance and interpretability (35). It can deeply perceive the relationship between entities in the process of modeling by graph-structured data. Vertexes and edges connecting vertexes together constitute graph structure. The vertexes are object entities, and the edges are the specific relationship between the entities. Specifically, several common agricultural products can be regarded as entities, and the correlation between them can be regarded as edges, which together constitute a kind of graph network. By introducing a neural computing structure, a GNN model for time series prediction can be constructed. Therefore, this study designs a graph neural network-based smart management for the agricultural products market (GNN-APM). The main highlights of this article can be summed up as follows:

- The internal complexity of agricultural products systems is investigated for further management.
- The GNN is employed to construct a time-series price prediction method for agricultural products systems.
- A case study is carried out to evaluate the performance of the proposed method on real-world scenes.

## 2. System Model

**Figure 1:**
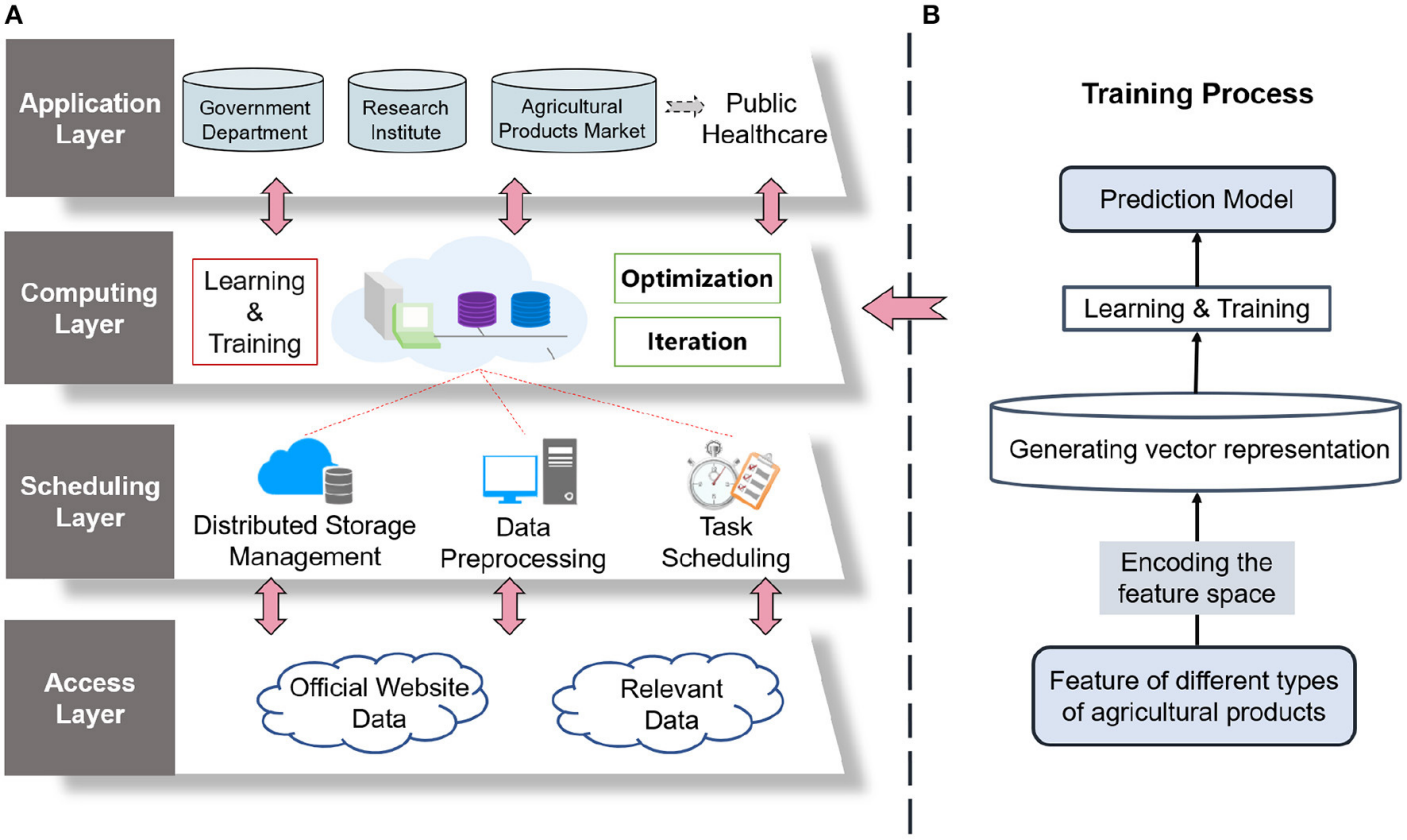
System model and overview of the designed graph neural network-based smart management for agricultural products market (GNN-APM). **(A)** architecture and **(B)** training process in computing layer.

The left part of Figure 1 illustrates the learning and training process inside GNN-APM. First, the feature space of different types of agricultural products is coded into vectorized representations. Then a prediction model is obtained by learning the data samples. As a necessity in our life, agricultural products have a strong correlation among various categories, and these correlations are very meaningful and worth exploring further. The complex interrelation between different categories affects their demand to a great extent. The introduction of the GNN model deeply excavates the correlation between different categories of agricultural products and builds a graph structure with agricultural products as the entity. Through analysis, the market conditions of agricultural products can be predicted and further intelligent management of public health can be realized.

Generalized to the problem scenario in this study, there are several types of agricultural products whose market conditions need to be forecasted. The learning algorithm of GNN-APM is shown in Algorithm 1. Types of agricultural products are viewed as the set of nodes, and their internal relations are regarded as the set of edges. Market conditions of agricultural products refer to the average market price in this research and will be updated temporally. Each time that the market condition is updated, is defined as a timestamp *t* which ranges from 1 to *M*. During each timestamp, market conditions of all the agricultural types are denoted as $V_{i}^{\left(t\right)}$, where *i* is the index number of agricultural products types. Inputting market conditions data of *M* timestamps, the main goal is to predict unknown market conditions data of following timestamps. Obviously, internal relations among nodes are likely to influence the tendency of market conditions. Thus, a relationship-aware sequential forecasting problem is formulated, and the GNN model is adopted to deal with such a problem.

**Algorithm 1:** The learning algorithm of GNN-APM.

Input: The market condition dataset: *D*; node set: *V*; number of agricultural products: *N*; total timestamp: *M*; learning rate: *l*; parameter set: Θ; penalty parameter: *p*;
1: initial *iter* = 1
2: repeat
3: for *t* ∈ [1, *M*] **do**
4: for *i* ∈ [1, *N*] **do**
5: compute $V_{i}^{\left(t\right)}=f(\theta (;;id(V_{i});\Theta )$;
6: compute $loss=\overset{N}{\underset{i=1}{\sum }}\overset{M}{\underset{t=1}{\sum }}\left[∥V_{i}^{\left(t\right)}-{\hat{V}}_{i}^{\left(t\right)}∥+p*∥\Theta {∥}_{F}^{2}\right]$;
7: compute the gradient of Θ according to *loss*;
8: update model parameters Θ according to their gradients and learning rate *l*;
9: end **for**
10: end **for**
11: *iter* = *iter*+1;
12: until convergence

## 3. Methodology

Graph convolution network (GCN), a typical GNN model, is utilized here to model correlated sample space. The GCN extends the convolution operation to non-Euclidean data with graph structure. It is a deep learning method for graph structured data. Graph data can naturally represent data structures in real life, such as traffic networks, communication networks, and social networks. In other words, it is the way to represent this kind of data format. Unlike image and text data, graph data has different local structures for each node. This is because the nodes in the graph represent the different entities in the network, and the edges that connect the nodes represent the relationships between the entities.

**Figure 2:**
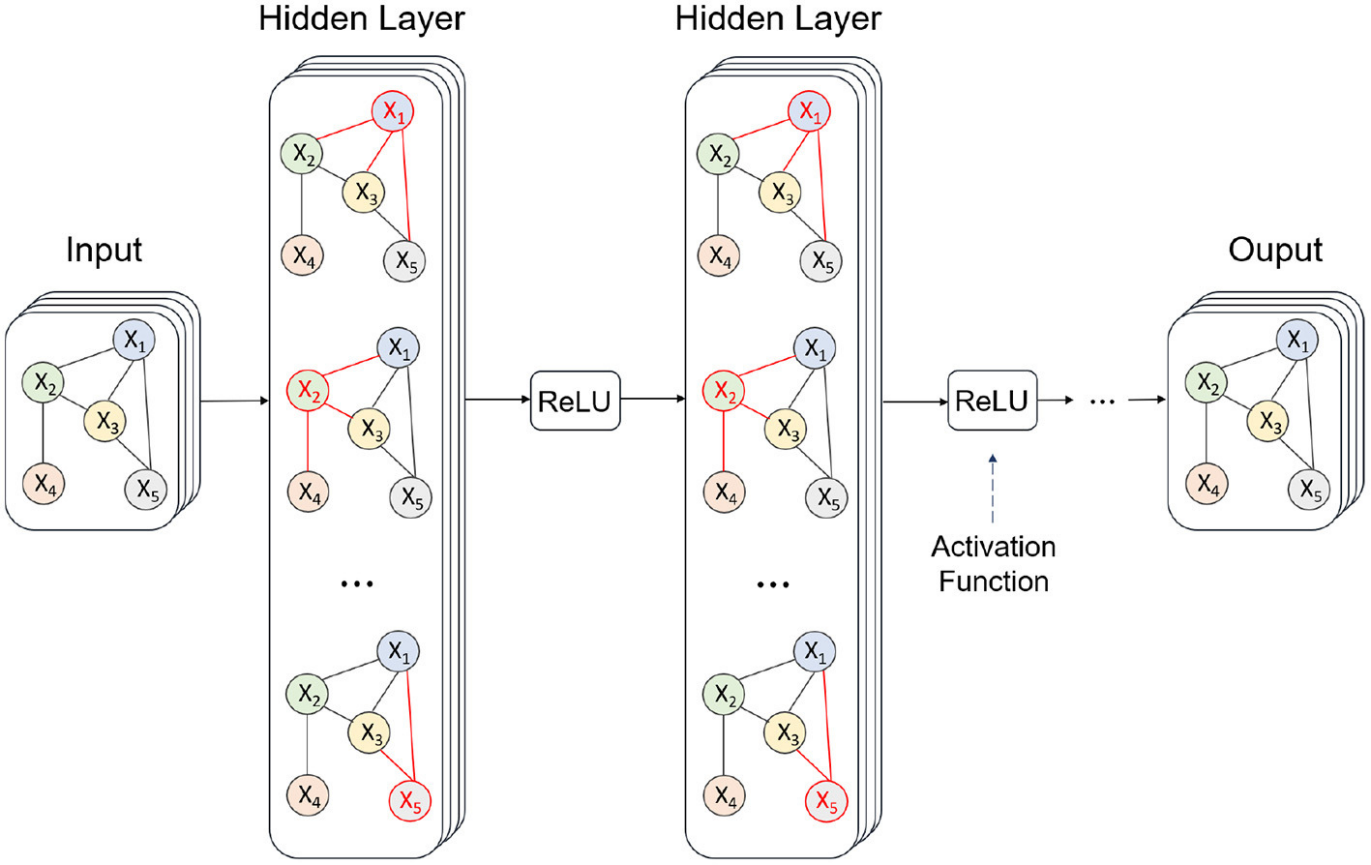
Workflow of the graph convolutional network employed for prediction.

As is shown in Figure 2, taking graph structure as input, GCN obtains new node representation through graph convolution operation on neighbor nodes of each node in the graph. Then, all nodes are pooled to obtain the representation of the whole graph. In particular, an undirected graph with nodes is defined as *G*(*V, E*), where *V* is the number of nodes and *E* is the edge between two nodes. Enumerating *i* from 1 to *N*, *v*_*i*_ constitutes the node set *V*. Let *j* denote the index number of nodes different from node *i*, edge *e*_*ij*_ constitutes all the edges between pairs of nodes. Additionally, all the edge states inside graph *G* are able to make up an adjacency matrix *A*.

There are two kinds of GCN methods: spectral method and spatial method. Spectral CNN is the first method to construct a convolutional neural network on the graph. This method uses the convolution theorem on the graph to define graph convolution from the spectral domain. Specifically, it uses the convolution theorem to define the graph convolution operator in each layer. Under the guidance of the loss function, it learns the convolution kernel by gradient backpropagation and builds neural networks by stacking multiple layers. The spectral method is more general in most time-series prediction problems and is selected as the main technique of this study.

The spectral GCN method derives from the Fourier transform (FT) theory which can transform signals of the time domain into signals of the frequency domain. After such transformation, complicated convolution operations of the time domain can be approximated as multiplication operations of the frequency domain. The FT and inverse Fourier transform (IFT) are defined as follows:


(1) $$\begin{array}{l} \hat{x}=FT\left(x\right)=U^{T}x \end{array}$$

(2) $$\begin{array}{l} x=IFT\left(x\right)=U\hat{x} \end{array}$$

where *U* is the eigenvector that approximates the FT to matrix computation. Thus, the graph convolution operation is defined as follows:


(3) $$\begin{array}{l} x{⊗}_{G}g=U\left(U^{T}x⊙U^{T}g\right) \end{array}$$

where *x* is the input, ⊗_*G*_ is the graph convolution operator, *g* is the core, and ⊙ is the harmand product operator. Introducing Laplacian eigenvector as a basis function, the input signal can be expanded as:


(4) $$\begin{array}{l} x\left[\begin{array}{c} x\left(1\right)\\ x\left(2\right)\\ \vdots \\ x\left(\gamma \right) \end{array}\right]=\left(u_{1},u_{2},\cdots \;,u_{\gamma }\right)\left[\begin{array}{c} \hat{x}\left(1\right)\\ \hat{x}\left(2\right)\\ \vdots \\ \hat{x}\left(\gamma \right) \end{array}\right] \end{array}$$

Expanding *g* with matrix forms and then substituting Equation (4) into Equation (3), the following formula can be deduced:


(5) $$\begin{array}{l} x{⊗}_{G}g=U\left[\begin{array}{c} ĝ\left(\lambda_{1}\right)\\ \ddots \\ ĝ\left(\lambda_{\gamma }\right) \end{array}\right] \end{array}$$

Letting *Chev*(λ_1_) denote first-order Chebyshev polynomials of λ_1_, the convolution operator *g* can be approximated as:


(6) $$\begin{array}{l} g_{\theta }=U\left[\begin{array}{c} Chev\left(\lambda_{1}\right)\\ \ddots \\ Chev\left(\lambda_{\gamma }\right) \end{array}\right] \end{array}$$

Among, the *Chev*(λ_γ_) can be represented as:


(7) $$\begin{array}{l} Chev\left(\lambda_{\gamma }\right)=\xi_{0}\Gamma_{0}\left(\lambda_{\gamma }\right)+\xi_{1}\Gamma_{1}\left(\lambda_{\gamma }\right) \end{array}$$

Hence, Equation (5) can be rewritten as the following formula:


(8) $$\begin{array}{l} x{⊗}_{G}g=\left[\alpha_{0}-\alpha_{1}\left(E^{-\frac{1}{2}}\cdot A\cdot E^{-\frac{1}{2}}\right)\right]x \end{array}$$

where *E* is the degree matrix, and α_0_ and α_1_ are parameters to be learned. For simplicity, it is supposed to set α_0_ = α_1_ = −θ. Therefore, the above equation can be rewritten as:


(9) $$\begin{array}{l} x{⊗}_{G}g=\left[\theta \left(E^{-\frac{1}{2}}\cdot A\cdot E^{-\frac{1}{2}}+D_{\eta }\right)\right] \end{array}$$

In order to facilitate searching for optimum, renormalization operation is conducted on the above formula:


(10) $$\begin{array}{l} E^{-\frac{1}{2}}\cdot A\cdot E^{-\frac{1}{2}}+D_{\eta }\approx E^{-\frac{1}{2}}\cdot Ã\cdot E^{-\frac{1}{2}} \end{array}$$

(11) $$\begin{array}{l} Ã\approx A+D_{\eta } \end{array}$$

(12) $$\begin{array}{l} {Ẽ}_{ij}\approx \underset{i}{\sum }{Ã}_{ij} \end{array}$$

where Ẽ_*ij*_ is the degree matrix of the *i*-th node, and $\underset{i}{\sum }{Ã}_{ij}$ is the number of edges between the *i*-th node and other nodes. The final expression of graph convolution operation can be represented as:


(13) $$\begin{array}{l} x{⊗}_{G}g=\theta \left(E^{-\frac{1}{2}}\cdot Ã\cdot E^{-\frac{1}{2}}\right)x \end{array}$$

At the *t*-th timestamp, the main input is related to the outputs of the previous several timestamps and the relation status among nodes. For the *i*-th node, its information state at the *t*-th timestamp can be represented as the following formula:


(14) $$\begin{array}{l} S_{i}^{\left(t\right)}=\beta \cdot O_{i}^{\left(t\right)}\cdot W_{S1}+\left(1-\beta \right)\cdot a_{i}\cdot W_{S2} \end{array}$$

where $O_{i}^{\left(t\right)}$ is a vector that records output values at previous several timestamps, *a*_*i*_ is a vector that records relation status between the *i*-th node and other nodes, β is a tuning parameter that adjusts the weight of two parts in Equation (14), and *W*_*S*1_ and *W*_*S*2_ are parameters to be learned. It is widely known that the internal of GCN is the information propagation process, as GCN emphasizes modeling of various dynamic or static relations. Accordingly, the representative vectors for node status can be also propagated to the following timestamps, which can be expressed as the following formula:


(15) $$\begin{array}{l} S_{i}^{\left(t+1\right)}=\sigma_{1}\left[{Ẽ}^{-\frac{1}{2}}\cdot S_{i}^{\left(t\right)}\cdot {Ẽ}^{-\frac{1}{2}}\cdot W_{S3}+b_{S1}\right] \end{array}$$

where *W*_*S*3_ and *b*_*S*1_ are parameters, and σ_1_(·) is the Reluctant Unit activation function represented as follows:


(16) $$\sigma_{1}\left(x\right)=\{\begin{array}{cc} 0, & x\leq 0\\ x, & x>0 \end{array}$$

Hence, the prediction result at the *t*-th timestamp can be calculated as the following formula:


(17) $$\begin{array}{l} V_{i}^{\left(t\right)}=\sigma_{1}\left[S_{i}^{\left(t\right)}\cdot W_{S4}+b_{S2}\right] \end{array}$$

where *W*_*S*4_ and *b*_*S*2_ are parameters. As for training, the following optimization objective can be formulated to search for the optimal parameters:


(18) $$\text{min}\sum_{i=1}^{N}\sum_{t=1}^{M}\left[∥V^{\left(t\right)}-{\hat{V}}^{\left(t\right)}∥+p\cdot ∥\Theta {∥}_{F}^{2}\right]$$

where *V*^(*t*)^ is the predicted result at the *t*-th timestamp, ${\hat{V}}^{\left(t\right)}$ is the real result at the *t*-th timestamp, Θ is the parameter set, *p* is the penalty parameter, and $∥\cdot {∥}_{F}^{2}$ is the Frobenius norm. Finally, the Adam optimization algorithm can be utilized to search optimal solution for Equation 18.

## 4. Setting of Case Study

To evaluate the GNN-APM designed in this study, real-world data is used here to set a simulative analysis situation. The real-world data was crawled from the official website of the Ministry of agriculture of China ^1^, including the market data of several key agricultural products from April 2019 to March 2021. The data demonstrates the average market price of agricultural products wholesale and contains five types of agricultural products. Market condition data for these agricultural products are updated once a week, and there are totally 96 weeks of data concerning the five types of agricultural products. Of all the five types of agricultural products, there are 10 kinds of node combinations, indicating 10 kinds of edges among these nodes. In other words, the adjacency matrix in this situation is a matrix with five columns and five lines, representing relation status between every two combinations of nodes.

During each round of simulations, these 10 groups of relations are randomly generated according to a Gaussian distribution whose mean is set to 0.5 and variance is set to 0.05. As for the ratio between training data and testing data, it is majorly set to 7:3 and 6:4. To quantify the error between prediction results and real results, two typical metrics are selected. They are mean absolute error (MAE) and root mean squared error (RMSE). In addition, two general prediction models are employed as baseline methods for comparison. Different from the designed GNN-APM in this study, the two baseline methods never take internal correlations among nodes into consideration. The two methods are the long short-term memory (LSTM) model and the multi-layer perceptron (MLP) model.

## 5. Results and Analysis

In this study, the whole simulative experiments are composed of three parts. First, the fluctuation tendency of the involved five agricultural product types is visualized using a curve diagram. Second, the prediction efficiency of the GNN-APM on five objects is compared with two baseline methods. Third, the robustness of the GNN-APM is tested by changing several parameter combinations.

### 5.1. Data Pre-processing

**Figure 3:**
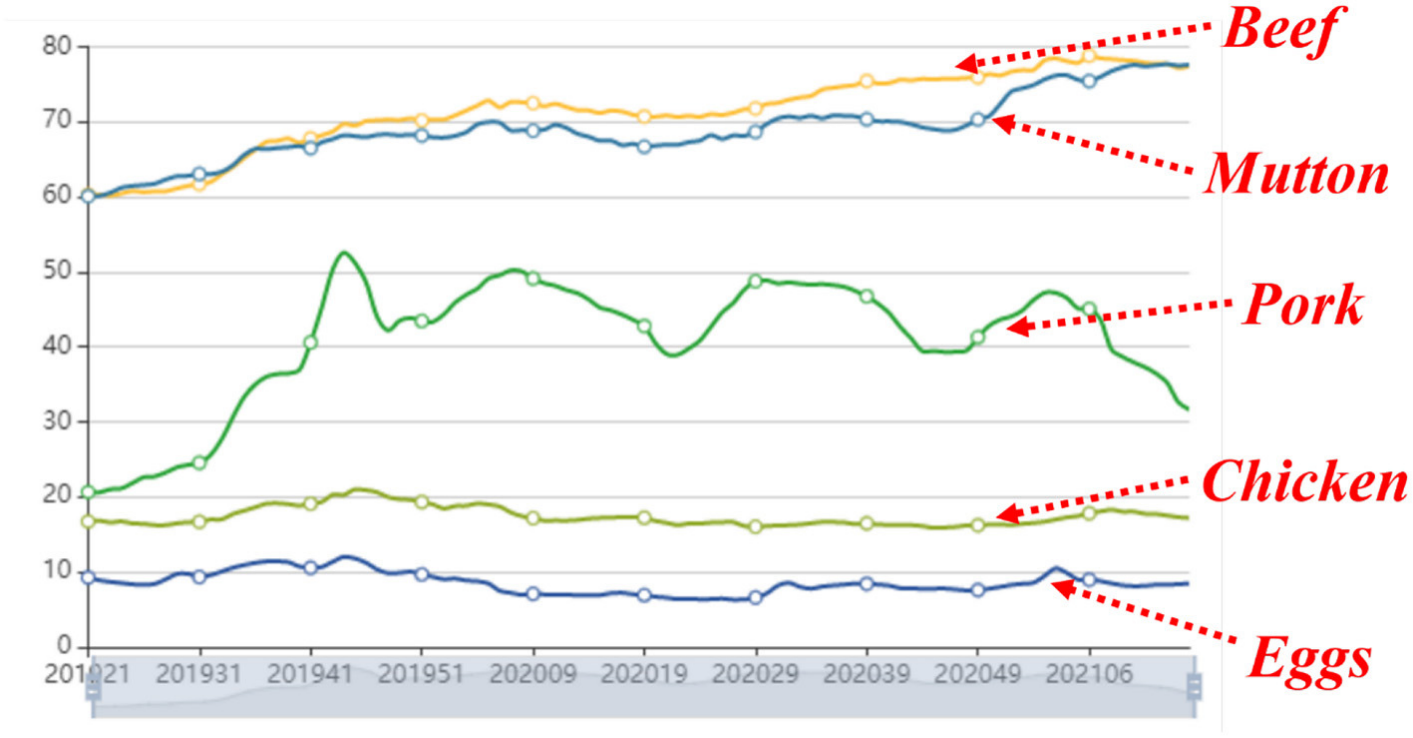
The total tendency of market conditions concerning five types of agricultural products.

Figure 3 visualizes the total tendency of market conditions for five types of agricultural products. It can be observed from the figure that five curves from the bottom to the top correspond to eggs, chicken, pork, mutton, and beef. During a long period about nearly 2 years, eggs and chicken remain relatively stable, mutton and beef show an ascending tendency, and the pork fluctuates frequently. These five types of agricultural products possess their own fluctuation tendencies and satisfy the assumption of diversity. And it can be also seen that the fluctuation tendency of pork has some effect on the other four types of agricultural products. Thus, the assumption that correlations exist among these types of agricultural products is reasonable.

### 5.2. Performance Assessment

Tables 1, 2, respectively give MAE results and RMSE results of these experimental methods when the proportion of training data ranges from 50 to 70% and the learning rate ranges from 0.001 to 0.002. Each of them has five lines and seven rows. The first two lines list the experimental setting, and the other lines present the experimental results of three methods. The first row lists three experimental methods, the second to the fourth rows present results under a learning rate of 0.001, the fifth to the seventh rows present results under a learning rate of 0.002. It can be observed from the two tables that MAE results and RMSE results of GNN-APM are below two other baseline methods, regardless of the proportion of training data and learning rate. This demonstrates that the performance of the GNN-APM is better than baseline methods.

**Table 1:** Mean absolute error (MAE) results when proportion of training data ranges from 50 to 70% and learning rate ranges from 0.001 to 0.002.

**Setting**	**Learning rate: 0.001**			**Learning rate: 0.002**		
	**50%**	**60%**	**70%**	**50%**	**60%**	**70%**
MLP	1.189	1.047	1.016	1.232	1.175	1.143
LSTM	1.116	1.052	1.044	1.139	1.091	1.107
GNN-APM	**0.964**	**0.927**	**0.875**	**0.989**	**0.903**	**1.016**

*The bold values correspond to results of the proposed GNN-APM method*.

**Table 2:** Root mean squared error (RMSE) results when the proportion of training data ranges from 50 to 70% and the learning rate ranges from 0.001 to 0.002.

**Setting**	**Learning rate: 0.001**			**Learning rate: 0.002**		
	**50%**	**60%**	**70%**	**50%**	**60%**	**70%**
MLP	1.664	1.458	1.373	1.761	1.663	1.589
LSTM	1.512	1.408	1.387	1.553	1.476	1.481
GNN-APM	**1.289**	**1.245**	**1.177**	**1.343**	**1.184**	**1.353**

*The bold values correspond to results of the proposed GNN-APM method*.

**Figure 4:**
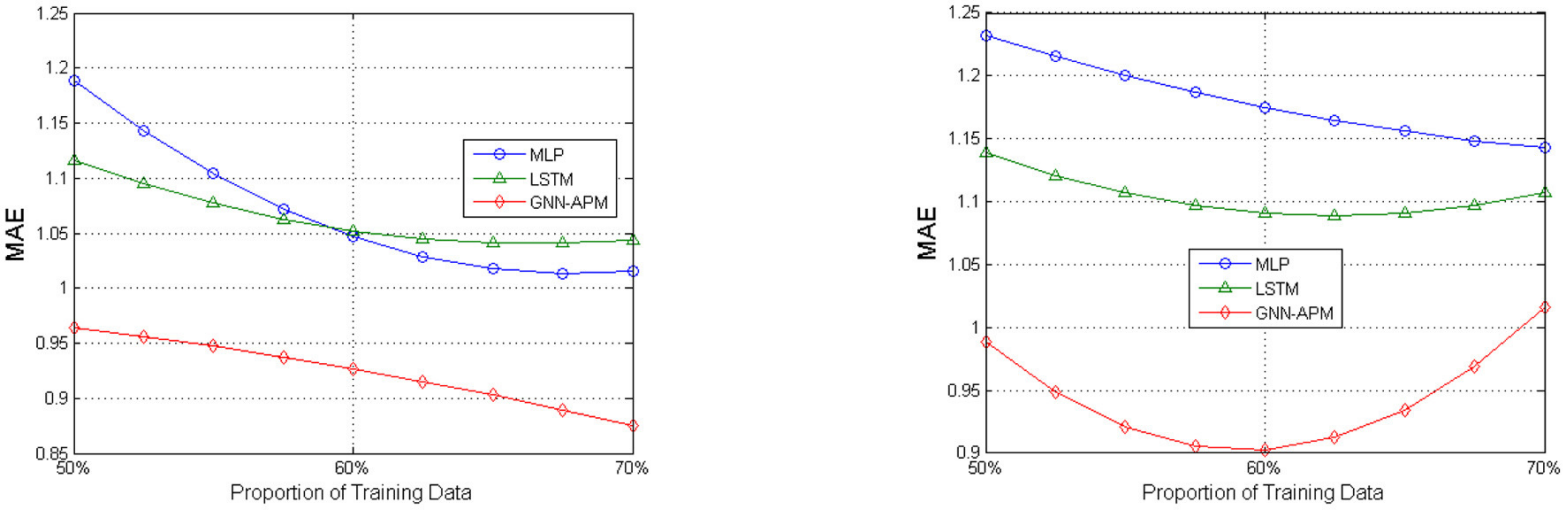
Average mean absolute error (MAE) results under two different learning rate values.

**Figure 5:**
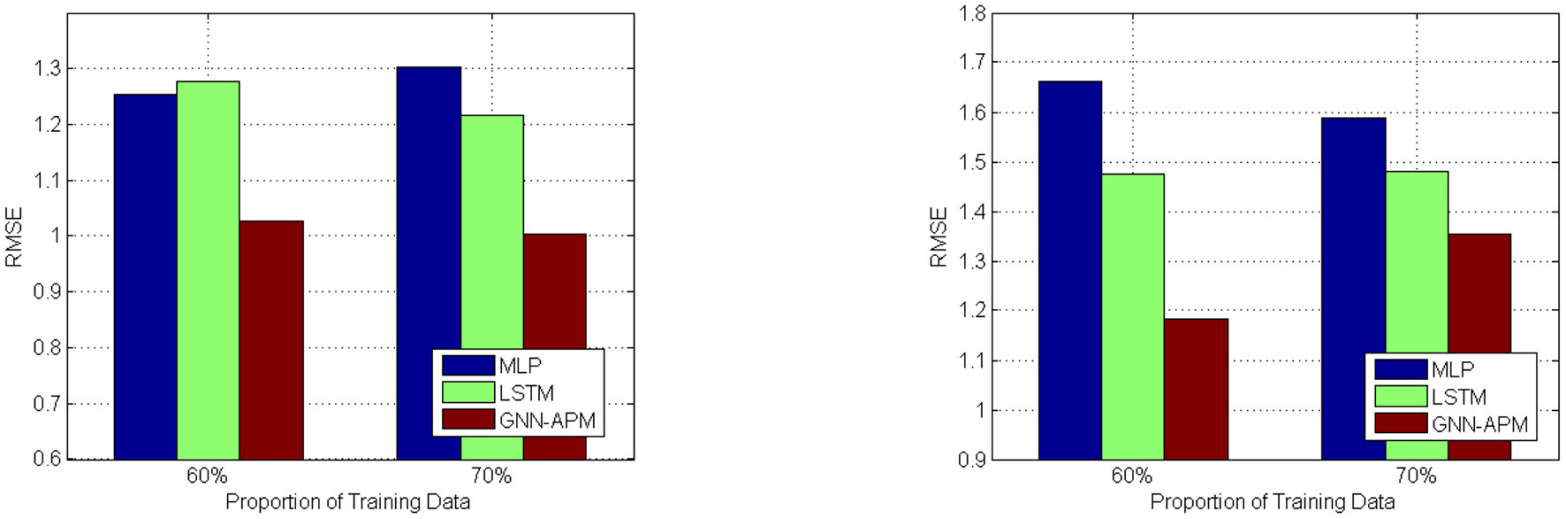
Average RMSE results under two different learning rate values.

Figures 4, 5 illustrates prediction efficiency with respect to using two metrics: MAE and RMSE. As there are totally five types of agricultural products involved, the MAE results and RMSE results are obtained as the mean value of prediction results on the five types. This figure has two subfigures, corresponding to MAE results and RMSE results. Among them, Figure 4 is the curve diagram and Figure 5 is the bar diagram. For the former, the X-axis demonstrates three kinds of training sizes and the Y-axis demonstrates values of MAE results. For the latter, only the two most typical training sizes are utilized for evaluation. Thus, it has two clusters of bars, corresponding to RMSE results under two training sizes. It is clearly observed that the GNN-APM is always endowed with better prediction efficiency compared with baseline methods. To sum up, this group of simulative experiments well demonstrates the good performance of the designed GNN-APM.

In order to visualize the tendency of MAE results and RMSE results under different experimental settings, some of the results are illustrated with the use of curve diagrams or bar diagrams. Figure 4 illustrates the MAE results of three methods under two different learning rate values: 0.001 and 0.002. It has two subfigures that correspond to results about two learning rate values. In each subfigure, the X-axis denotes the proportion of training data ranging from 50 to 70%, and the Y-axis denotes values of MAE results. Figure 5 illustrates RMSE results of three methods when the training data size is set to 60 and 70%. This is because it can be seen from previous experiments that result under the two training data sizes are relatively better. It has two subfigures that correspond to two learning rate values. In each subfigure, the X-axis denotes two training sizes, and the Y-axis denotes values of RMSE results. It can be observed from these figures that values of GNN-APM are obviously below the other two methods and that values show descending tendency when the proportion of training data increases. Such results demonstrate the improvement process of methods with being trained more sufficiently. These figures show a better performance tendency of GNN-APM compared with two other baseline methods.

### 5.3. Parameter Sensitivity

**Figure 6:**
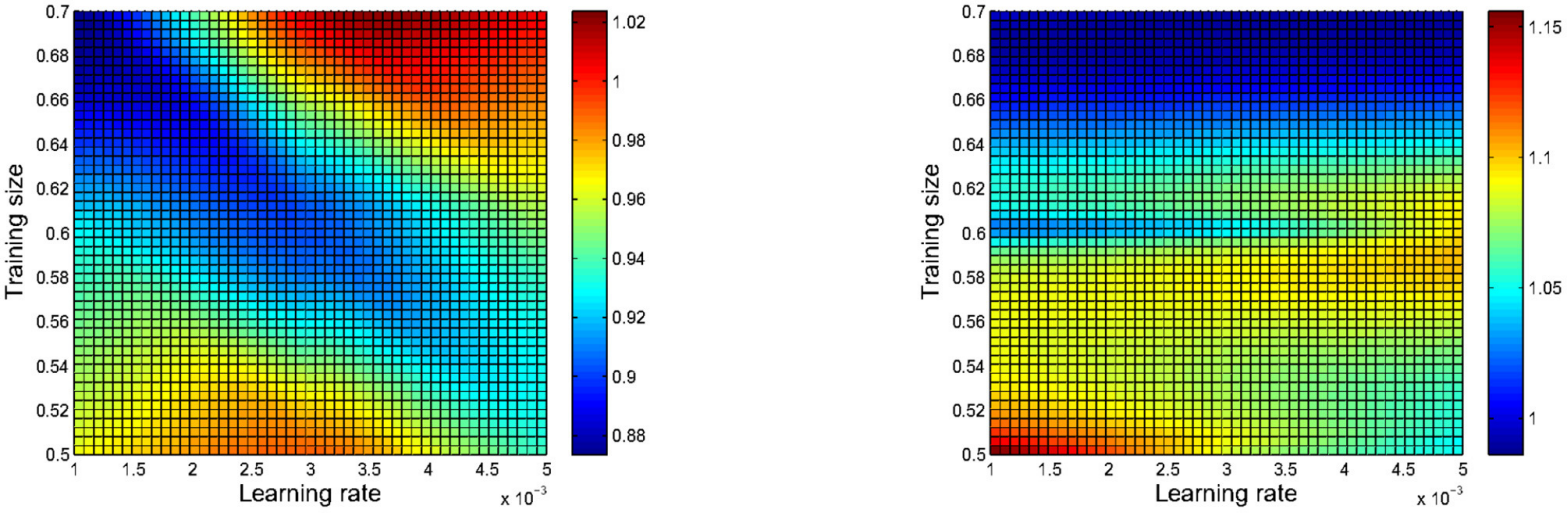
Parameter sensitivity results of the designed GNN-APM concerning MAE and RMSE.

Besides, it is also expected to explore parameter sensitivity of the GNN-APM, and relevant simulative results are illustrated in Figure 6. During this group of experiments, the GNN-APM is not compared with baseline methods and just performance of itself is explored. Figure 6 has two subfigures, corresponding to sensitivity results using two different metrics: MAE and RMSE. Inside each subfigure, the X-axis denotes the change of learning rate, and the Y-axis denotes the change of training size. In the middle square area, the color depth indicates the different values of evaluation metrics. As the two subfigures are heatmaps, the color depth degree inside figures is able to indicate values of metrics. Each subfigure includes a squared area, gentle color change inside it indicates that the performance of GNN-APM fluctuates not heavily. It can be objectively found that color fluctuation in both two subfigures seems quite gentle, revealing that the GNN-APM is not susceptible to parameter change. In other words, the GNN-APM is always able to remain stable, no matter how the key parameters change. This group of simulative results well prove that the GNN-APM possesses proper robustness.

## 6. Conclusion

Agriculture has been viewed as the most fundamental industry since ancient times. Nowadays, E-commerce is an important sales channel of agricultural products. To better manage and schedule the supply of agricultural products, dynamic price prediction for agricultural products in the E-commerce market is of great significance. To overcome the shortcomings of existing research studies, this article proposes a deep learning-based price prediction model for agricultural products in the E-commerce market. In particular, the most typical GCN is utilized to establish a time-series prediction model for the dynamic price of agricultural products. In addition, the whole simulative experiments are composed of three parts. First, the fluctuation tendency of the involved five agricultural product types is visualized using a curve diagram. Second, the prediction efficiency of the GNN-APM on five objects is compared with two baseline methods. Third, the robustness of the GNN-APM is tested by changing several parameter combinations.

Nowadays, data mining and data management for many industries are gradually approaching the application of the Internet of Things (IoT), yielding such as mobile IoT (36, 37), financial IoT, medical IoT (38), cloud-assisted IoT (39), vehicular IoT (40), and industrial IoT (41, 42). As is known to all, the IoT is a kind of effective tool or platform to integrate multi-domain data and schedule business flows. To realize more effective scheduling management of the agricultural product market, designing an integrated microservice IoT platform that is embedded with robust artificial intelligence algorithms (43), is in urgent demand to deal with many disturbing issues in various industries. Thus, for future outlook, the authors plan to deeply investigate optimal scheduling and management schemes for the agricultural product market with the use of novel IoT-related technologies.

## Data Availability Statement

Publicly available datasets were analyzed in this study. This data can be found at: http://www.moa.gov.cn/.

## Author Contributions

WY contributed significantly to theoretical analysis and manuscript preparation. ZZ performed the experiments and handled funding details. QZ contributed to the conception of the study and model formulation. GZ performed the data analyses and data visualization. QH provided many promising insights during the revision process. QL helped perform the analysis with constructive discussions. All authors contributed to the article and approved the submitted version.

## Funding

This study was supported in part by Beijing Natural Science Foundation (No. 4202014), Natural Science Foundation of China (61873027), in part by Humanity and Social Science Youth Foundation of Ministry of Education of China (No. 20YJCZH229), in part by the Open Project Program of National Engineering Laboratory for Agri-Product Quality Traceability (AQT-2020-YB8), and in part by Hubei Natural Science Foundation (No. 2021CFB156).

## Conflict of Interest

The authors declare that the research was conducted in the absence of any commercial or financial relationships that could be construed as a potential conflict of interest.

## Publisher's Note

All claims expressed in this article are solely those of the authors and do not necessarily represent those of their affiliated organizations, or those of the publisher, the editors and the reviewers. Any product that may be evaluated in this article, or claim that may be made by its manufacturer, is not guaranteed or endorsed by the publisher.

## Footnotes

^1^ http://www.moa.gov.cn/
